# External validation of three atherosclerotic cardiovascular disease risk equations in rural areas of Xinjiang, China

**DOI:** 10.1186/s12889-020-09579-4

**Published:** 2020-09-29

**Authors:** Yunxing Jiang, Rulin Ma, Heng Guo, Xianghui Zhang, Xinping Wang, Kui Wang, Yunhua Hu, Mulatibieke Keerman, Yizhong Yan, Jiaolong Ma, Yanpeng Song, Jingyu Zhang, Jia He, Shuxia Guo

**Affiliations:** 1 Department of Public Health, Shihezi University School of Medicine, North 2th Road, Shihezi, Xinjiang, 832000 China; 2 The First Affiliated Hospital of Shihezi University Medical College, Shihezi, Xinjiang, 832000 China; 3 Department of Pathology and Key Laboratory of Xinjiang Endemic and Ethnic Diseases (Ministry of Education), Shihezi University School of Medicine, Shihezi, Xinjiang, 832000 China

**Keywords:** Atherosclerotic, Cardiovascular diseases, Primary prevention, Risk equations, External validation

## Abstract

**Background:**

To externally validate the Prediction for ASCVD Risk in China (PAR) risk equation for predicting the 5-year atherosclerotic cardiovascular disease (ASCVD) risk in the Uyghur and Kazakh populations from rural areas in northwestern China and compare its performance with those of the pooled cohort equations (PCE) and Framingham risk score (FRS).

**Methods:**

The final analysis included 3347 subjects aged 40–74 years without CVD at baseline. The 5-year ASCVD risk was calculated using the PAR, PCE, and FRS. Discrimination, calibration, and clinical usefulness of the three equations in predicting the 5-year ASCVD risk were assessed before and after recalibration.

**Results:**

Of 3347 included subjects, 1839 were female. We observed 286 ASCVD events in within 5-year follow-up. All three risk equations had moderate discrimination in both men and women. C-indices of PAR, PCE, and FRS were 0.727 (95% CI, 0.725–0.729), 0.727 (95% CI, 0.725–0.729), and 0.740 (95% CI, 0.738–0.742), respectively, in men; the corresponding C-indices were 0.738 (95% CI, 0.737–0.739), 0.731 (95% CI, 0.730–0.732), and 0.761 (95% CI, 0.760–0.762), respectively, in women. PCE, PAR and FRS substantially underestimated the 5-year ASCVD risk in women by 70, 23 and 51%, respectively. However, PAR and FRS fairly predicted the risk in men and PAR was well calibrated. The calibrations of the three risk equations could be changed by recalibration. The decision curve analyses demonstrated that at the threshold risk of 5%, PCE was the most clinically useful in both men and women after recalibration.

**Conclusions:**

All three risk equations underestimated the 5-year ASCVD risk in women, while PAR and FRS fairly predicted that in men. However, the results of predictive performances for three risk equations are inconsistent, more accurate risk equations are required in the primary prevention of ASCVD aiming to this Uyghur and Kazakh populations.

## Background

Atherosclerotic cardiovascular disease (ASCVD), including ischemic heart disease and ischemic stroke, is the leading cause of mortality and disease burden in the world and has been an important public health concern globally [1]. ASCVD incidence risk is associated with factors such as smoking or blood cholesterol, which are potentially modifiable by lifestyle changes or medication use [2]. Timely identification of individuals at high risk of having ASCVD events through risk assessment tools can effectively promote the primary prevention of ASCVD.

The recently released Guideline on the Assessment and Management of Cardiovascular Risk in China [3] recommended calculating ASCVD risks to identify those who at high risk of ASCVD using the newly developed Prediction for ASCVD Risk in China (PAR) risk equation [4], which were generated in general Chinese population. After the development of the PAR, it has been undergone an external validation in a rural northern Chinese population, however, the performance of the PAR in predicting 5-year ASCVD risk was just moderate [5].

The Xinjiang province is a typically multi-ethnic area, which is situated in the northwest of China approximately 2000 miles from Beijing. The Uyghur and Kazakh ethnic groups account for 45.84, 6.50% of the total population in Xinjiang respectively, and they have similar genetic backgrounds to Caucasians. The incidence rate of ASCVD is high among Uyghurs and Kazakhs due to their unique lifestyle, dietary habits, and genetic characteristics. Therefore, it is crucial to identify high-risk individuals using ASCVD risk assessment tools in the prevention of ASCVD. However, there has not been an ASCVD risk prediction model specially targeted at this population.

External validation is a crucial step to determine if a developed risk score can be used to guide the prevention and clinical decision making of ASCVD before applying to a population not used in the risk score’s derivation [6]. The newly developed the China-Population Attributable Risk equations (PAR) has not been validated in this population and whether this equation can be used to guide ASCVD prevention remains unclear.

Consequently, we designed this study to validate the PAR and compare its performance in predicting individual 5-year ASCVD risks against the Pooled Cohort equations (PCE) for White [7] recommended by The American College of Cardiology (ACC) and American Heart Association (AHA) guideline and the Framingham CV risk equation (FRS) [8] using the data from this independent and external Uyghur and Kazakh population in Xinjiang, China.

## Methods

### Study population

**Fig. 1:**
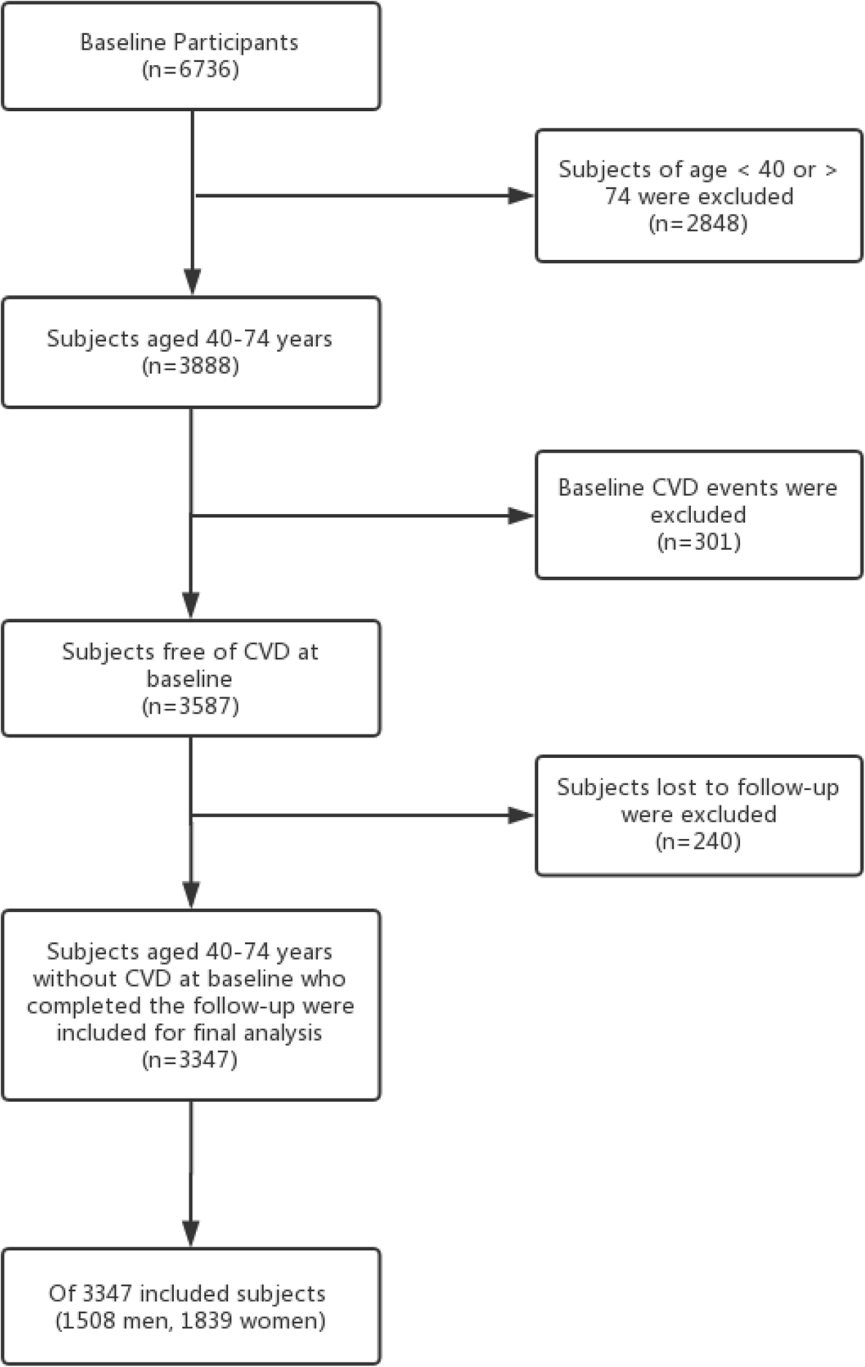
Flow diagram of subjects included in this study. CVD, Cardiovascular Disease; ASCVD, Atherosclerotic Cardiovascular Disease

The prospective cohort used in this study was designed to analyze metabolic syndrome and factors that predict the risk of CVD in a multi-ethnic rural area in China. Multistage (prefecture-county-township-village) stratified cluster random sampling was employed to choose participants. Firstly, we chose two representative prefectures (Kashi, Yili) based on the population, ethnicity, geography, economic and cultural development level, respectively in Xinjiang. Second, we randomly selected one county in each prefecture and one township from each county. Finally, a stratified sampling method was used to select the corresponding villages in each township [9]. A total of 6736 adults aged ≥18 years from Xinyuan and Jiashi County in China who had resided in the village for at least 6 months were enrolled in this study between April 2010 and December 2012 and followed for > 5 years on average by the end of December 2017. We restricted the analysis to participants aged 40–74 years without a history of cardiovascular disease (CVD) at baseline to directly compare the three risk assessment tools in predicting the 5-year atherosclerotic cardiovascular disease (ASCVD) risk. Based on the inclusion and exclusion criteria of the present study **(**Fig. 1**)**, 3347 subjects aged 40–74 years were included in the final analyses.

### Assessment of risk factors

Participants who provided informed consent underwent a face-to-face interview using a standard questionnaire to collect information on sociodemographic characteristics, medical history, and lifestyle habits during field investigation. Current cigarette smoking status was self-reported by participants. Family history of ASCVD was defined as myocardial infarction (MI) or stroke history in at least a parent or sibling. Treated hypertension was defined as hypertension with regular use of antihypertensive medications in the 2-week period before the interview. All participants lived in rural areas of Xinjiang, Northwestern China.

Following the interview, the participants were examined, and anthropometric measurements were obtained by trained health professionals. Waist circumference was defined as the midpoint between the lower rib and upper margin of the iliac crest at minimal respiration, as measured by a nonelastic ruler tape with an insertion buckle at one end to the nearest 0.1 cm. Blood pressure was measured in triplicate after a 5-min seated rest using an electronic sphygmomanometer, and the average of the measurements was obtained as the blood pressure of the individual. A 5-mL fasting blood sample was collected from each subject. The serum glucose, high-density lipoprotein cholesterol, and triglycerides were examined by a modified hexokinase enzymatic method using an Olympus AV2700 Biochemical Automatic Analyzer (Olympus, Japan) in the Biochemistry Laboratory of the First Affiliated Hospital of Shihezi University School of Medicine. Diabetes was defined as a fasting glucose level of ≥126 mg/dL in participants or self-report of a previous diagnosis of diabetes with current use of insulin or oral hypoglycemic medications.

### Assessment of outcome

An incident ASCVD event, the primary endpoint of this study, was defined as the first diagnosis of nonfatal acute MI or coronary heart disease (CHD) death or fatal or nonfatal stroke. Acute MI was defined as an increase in biochemical markers of myocardial necrosis, accompanied by ischemic symptoms, pathological Q waves, ST-segment elevation or depression, or coronary intervention [10]. CHD death included all fatal events due to MI or other coronary deaths. Stroke was classified as an ischemic or hemorrhagic attack. If the same type of ASCVD event occurred more than once, the first occurrence was considered the end event. The events in the study cohort during follow-up were identified from health insurance claims, patients’ hospital medical records, death registries from the morbidity and mortality surveillance system, and questionnaire responses. We conducted three follow-ups in 2013, 2016 and 2017, respectively. The questionnaire responses were acquired by professional investigators during a face-to-face visit. We usually follow up the subjects in November. First of all, we would record the basic demographic information and follow-up time in the questionnaire. If the subject died during the follow-up period, their family members were asked about the time of death, the place of death and the cause of death, and then the information was checked with the information obtained from the cause of death monitoring system. If the subjects survived, they would be asked whether they were hospitalized, and the reasons and time of hospitalization, and then the information would be verified with medical insurance data and medical record information to record their hospitalization diagnosis.

### Statistical analyses

Data were analyzed using SPSS version 17.0 for Windows (SPSS Inc., Chicago, IL, USA) and R software (version 3.2.3; https://www.r-project.org/). Continuous variables were summarized as sex-specific mean (Standard Deviation, SD) and analyzed using the t-test. Categorical variables were presented as numbers or percentages and analyzed using the chi-square test. Because this study is ongoing and has not yet completed the 10-year follow-up, we can only calculate observed and predicted ASCVD risks at 5 years. The 5-year baseline survival for PAR were calculated based on the reported 5-year Kaplan-Meier ASCVD incidence rate from derivation cohorts (S_0_(t) = exp. (− 5-year Kaplan-Meier ASCVD incidence rate)) [4, 11]. There was no reported 5-year Kaplan-Meier CVD incidence rate for the FRS, so the baseline survival was estimated as S_0_(t) = exp. (− 10-year Kaplan-Meier CVD incidence rate/2). The five-year survival rate of PCE was obtained from supplementary materials of Muntner, et al. [11]. Details in Additional file 1: Table S1.

For direct comparison, we used ASCVD as the outcome for all three risk equations. The predicted 5-year ASCVD risk for every participant in this cohort was calculated with the China-Population Attributable Risk equations (PAR) [4] and pooled cohort equations for White (PCE) [7] for ASCVD and the Framingham risk scores for CVD [8]. Details of the three risk equations are presented in Additional file 1: Table S2. The observed 5-year ASCVD risks were calculated using the Kaplan-Meier estimate [12].

Predictive performances of the three risk equations in predicting ASCVD endpoints for this population were evaluated by examining measures of calibration, discrimination, and clinical utility separately in men and women.

Discrimination is the ability of a risk equation to distinguish between individuals who have an ASCVD event during the follow-up and those who do not. Discrimination of each ASCVD risk equation was assessed using the C-index for survival data [13]. We also calculated the D statistic [14] and R^2^ statistic [15], which is explained variation measures. High R^2^ values indicate better discrimination.

Calibration refers to how accurately the predicted 5-year risk of ASCVD agrees with the observed 5-year risk. Calibration was assessed graphically by plotting the predicted 5-year risk against the observed risk of incident ASCVD events before and after recalibration, grouped according to deciles of predicted probabilities. A Greenwood-Nam-D’Agostino (GND) chi-square statistic (χ^2^) suitable for survival data was calculated [16], and a *P*-value > 0.05 indicates good calibration. Moreover, we also calculated the integrated Brier score for survival data, which measures the predictive accuracy of each ASCVD risk equation, and a lower score represents higher accuracy [17].

Because the characteristics of participants and the prevalence of ASCVD in our cohort were different from those in the derivation cohorts of PCE and PAR, recalibration of the risk equations was necessary before its application to this external population. After the initial evaluation of predictive performance, PCE and PAR were recalibrated by replacement with the mean values of the risk factors and average incidence rates at baseline in our cohort [18]. This process did not change the coefficients for risk factors. FRS has a broader definition of endpoints, so we did not recalibrated FRS.

To compare the clinical utilities of the PCE and PAR, we conducted decision curve analysis (DCA) after recalibration and calculated the net benefit of each equation in predicting the 5-year ASCVD risk [19].

To directly compare the recalibrated PCE and PAR, we calculated the integrated discrimination improvement (IDI), continuous net reclassification improvement (cNRI) and categorical net reclassification improvement (NRI) [20]. The recommended clinically useful cutoff points at 10 years were 5 and 10%, so in the reclassification tables, threshold probabilities of 2.5 and 5% were used for comparison of three risk equations in predicting 5-year ASCVD risk [21]. The study was conducted according to the Transparent Reporting of a multivariable prediction model for Individual Prognosis Or Diagnosis guidelines (TRIPOD) [22], and all analyses were performed separately in men and women.

## Results

### Baseline characteristics

Following the flowchart (Fig. 1), 3347 subjects aged 40–74 years were included in the final analysis and followed for a median of 7.05 years (IQR, 5.68–7.08). Of these subjects, 1839 (54.94%) were female. The mean ages were 52.10 (SD, 8.92) years and 53.83 (SD, 9.40) years in women and men, respectively. As expected, women had lower blood pressure, a lower prevalence of diabetes, and smoked less frequently. Additional clinical characteristics are shown in Table 1. During the follow-up, 286 subjects (137 men and 149 women) were diagnosed with ASCVD, and the 5-year observed risk of ASCVD was 9.1% (95% CI, 7.8–10.7%) in men and 8.4% (95% CI, 7.2–9.4%) in women.

**Table 1:** Baseline characteristics of study subjects in this validation set

Characteristics	Women (*n* = 1839)	Men (*n* = 1508)
Age, mean (SD), y	52.10 (8.92)	53.83 (9.40)
Waist circumference, mean (SD), cm	84.26 (10.70)	86.95 (10.15)
SBP, mean (SD), mmHg	131.29 (23.06)	134.34 (22.57)
DBP, mean (SD), mmHg	82.05 (14.43)	84.36 (14.18)
Total cholesterol, mean (SD), mg/dL	184.92 (33.47)	183.40 (33.26)
HDL-C, mean (SD), mg/dL	50.94 (11.80)	49.11 (9.84)
Family history of ASCVD, No. (%)	67 (3.64)	48 (3.18)
Diabetes, No. (%)	122 (6.63)	143 (9.48)
Anti-hypertension medications use, No. (%)	256 (13.92)	50 (3.32)
Current smoker, No. (%)	238 (12.94)	632 (41.91)
Total person years	10,800.41	9231.16
5-year Kaplan-Meier ASCVD rate (%)	8.4	9.1
Incidence of ASCVD events within 5 years	149	137

Abbreviations: *SD* Standard Deviation, *SBP* Systolic blood pressure, *DBP* Diastolic blood pressure, *HDL-C* High density lipoprotein cholesterol, *ASCVD* Atherosclerotic Cardiovascular Disease

### Discrimination and calibration

The predicted 5-year risks were calculated using published equations for these three models. The distributions of the predicted risks varied considerably between the three models in men and women as shown in Additional file 2**:** Fig. S1. The average estimated risks with the PCE, PAR, and FRS were 2.5, 6.4, and 4.1%, respectively, in women and 4.7, 9.6, and 9.7%, respectively, in men **(**Table 2**)**.

**Table 2:** Discrimination and calibration statistics for predicted 5-year risk of ASCVD by PCE, PAR and FRS

	PCE	PAR	FRS
**Women**			
C statistics (95%CI)	0.738 (0.703, 0.773)	0.731 (0.696, 0.766)	0.761 (0.728, 0.794)
D statistics	1.259 (1.253,1.265)	1.260 (1.254,1.266)	1.427 (1.421,1.433)
R^2^ statistics (%)	27.46 (27.27, 27.65)	27.50 (27.31, 27.69)	32.72 (32.54, 32.91)
Brier Score ^a^	0.0491	0.0486	0.0487
Greenwood-Nam-D’agostino (GND) calibration χ^2 c^	86.26	13.50	48.13
*P* value for GND test	0.000	0.009	0.000
Observed events ^b^	153.94	153.94	153.94
Predicted events	46.11	118.09	74.86
P/O	0.30	0.77	0.49
Average predicted risk (%)	2.5	6.4	4.1
Average observed risk (%)	8.4	8.4	8.4
Proportion of predicted risk> 5% (%)	16.51	54.05	29.31
**Men**			
C statistics (95%CI)	0.727 (0.689, 0.766)	0.727 (0.684, 0.770)	0.740 (0.703, 0.777)
D statistics	1.293 (1.286,1.300)	1.301 (1.294,1.308)	1.355 (1.348,1.362)
R^2^ statistics (%)	28.54 (28.32,28.76)	28.79 (28.57,29.01)	30.48 (30.26,30.70)
Brier Score a	0.0525	0.0523	0.0529
Greenwood-Nam-D’agostino (GND) calibration χ^2 d^	39.86	4.11	13.58
*P* value for GND test	0.000	0.534	0.019
Observed events ^b^	137.9	137.9	137.9
Predicted events	70.87	144.72	145.8
P/O	0.51	1.05	1.06
Average predicted risk (%)	4.7	9.6	9.7
Average observed risk (%)	9.1	9.1	9.1
Proportion of predicted risk> 5% (%)	55.74	49.92	57.31

Abbreviations: *P* Predicted events, *O* Observed events, *PCE* Pooled Cohort Risk Equations, *PAR* China-PAR risk equation, *FRS* Framingham Risk Score 2008

^a^Lower score indicates better accuracy of risk estimates;

^b^Adjusted using Kaplan-Meier method;

^c^Deciles were set as 5 to ensure that each decile contained at least 5 events

^d^Deciles were set as 6 to ensure that each decile contained at least 5 events

The discrimination and calibration performance metrics for PCE, PAR, and FRS are presented in Table 2. The C-indices of PAR, PCE, and FRS were 0.727 (95% CI, 0.689, 0.766), 0.727 (95% CI, 0.684, 0.770), and 0.740 (95% CI, 0.703, 0.777), respectively, in men; the corresponding C indices were 0.731 (95% CI, 0.703, 0.773), 0.738 (95% CI, 0.696, 0.766), and 0.761 (95% CI, 0.728, 0.794), respectively, in women. In women, the D discrimination statistic (a higher score indicates better discrimination) for FRS was higher than that for PCE and PAR, while the D discrimination statistic for FRS was higher than that for PCE and PAR in men. The R_D_^2^ proposed by Royston and Sauerbrei [15] as an explained variation measure for FRS in women was 5% higher than those for PCE and PAR, whereas the R_D_^2^ for PCE in men was similar to that of PAR and lower than that of FRS. We used the integrated Brier score to compare the prediction accuracy of risk equations, and a lower Brier score indicated greater accuracy of a risk equation. The Brier score for PCE was 0.0491 and was higher than that for PAR (0.0486) and FRS (0.0487) in women. The PAR had the lowest Brier score (0.0523), compared with PCE (0.0525) and FRS (0.0529) in men.

**Fig. 2:**
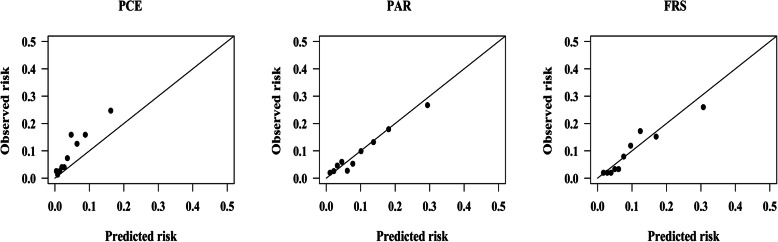
Calibration plots for the PCE, PAR and FRS in men before recalibration. PCE, Pooled Cohort Risk Equations; PAR, China-PAR risk equation; FRS, Framingham Risk Score 2008. GND test chi-square statistics for risk equations: PCE: 39.86; *P* < 0.001. PAR: 4.11; *P* = 0.534. FRS: 13.58; *P* = 0.019

**Fig. 3:**
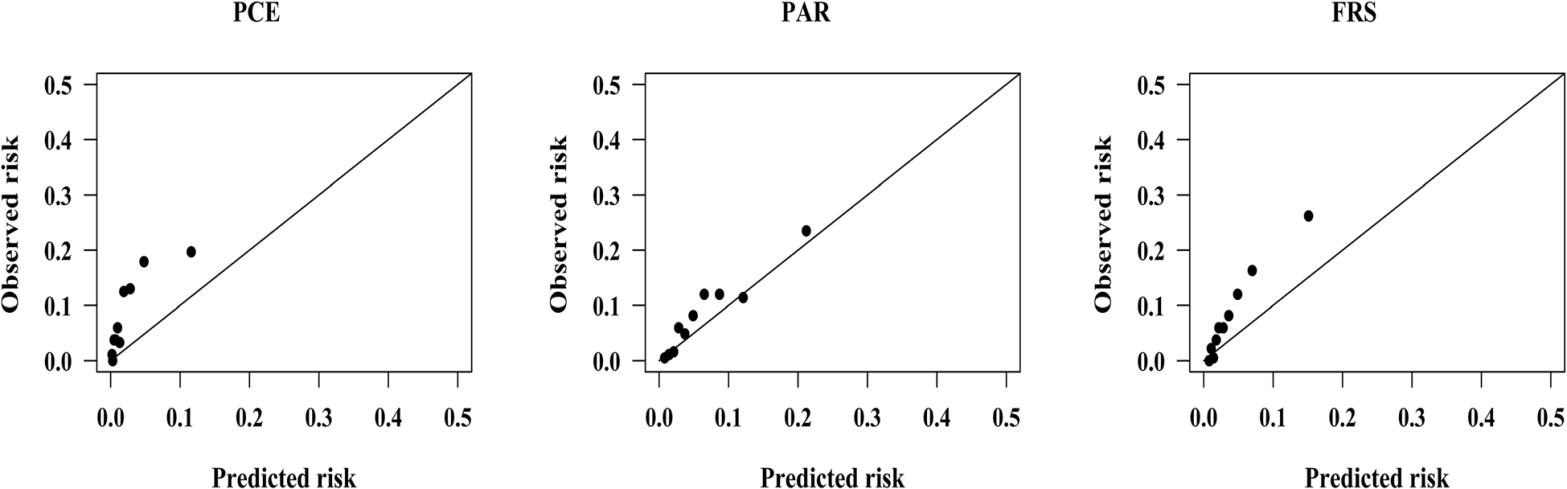
Calibration plots for the PCE, PAR and FRS in women before recalibration. PCE, Pooled Cohort Risk Equations; PAR, China-PAR risk equation; FRS, Framingham Risk Score 2008. GND test chi-square statistics for risk equations: PCE: 86.26; *P* < 0.001. PAR: 13.50; *P* = 0.009. FRS: 48.13; *P* < 0.001

The observed ASCVD events (adjusted using Kaplan-Meier method) was 137.90, while the predicted ASCVD events for PCE, PAR, and FRS was 70.87, 144.72, and 145.80, respectively, in men, giving a Predicted events/Observed events (P/O) of 0.51, 1.05, and 1.06, as shown in Table 2. The calibration plots (Fig. 2) show that FRS and PAR slightly overestimated the ASCVD risk, while PCE systematically underestimated the 5-year ASCVD risk in men by nearly 50%. The PCE, PAR and FRS substantially underestimated ASCVD risk by approximately 70, 23 and 51%, respectively, in women. The PAR was well calibrated (GND test χ^2^ = 4.11, *P* = 0.534). Calibration plots of PCE, PAR, and FRS in women are shown in Fig. 3**.**

### Recalibration of risk equations

**Fig. 4:**
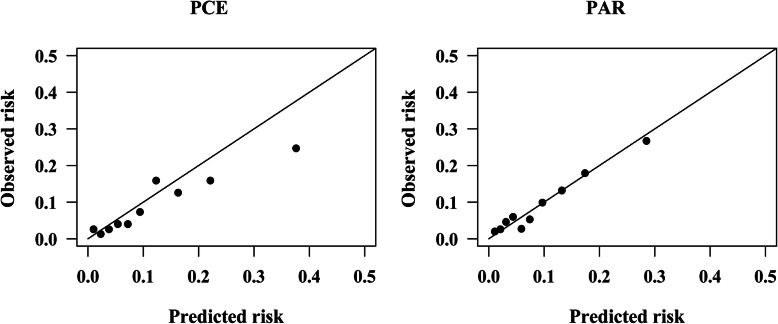
Calibration plots for the PCE and PAR in men after recalibration. PCE, Pooled Cohort Risk Equations; PAR, China-PAR risk equation; GND test chi-square statistics for risk equations: PCE: 26.74; *P* < 0.001. PAR: 3.47; *P* = 0.628

**Fig. 5:**
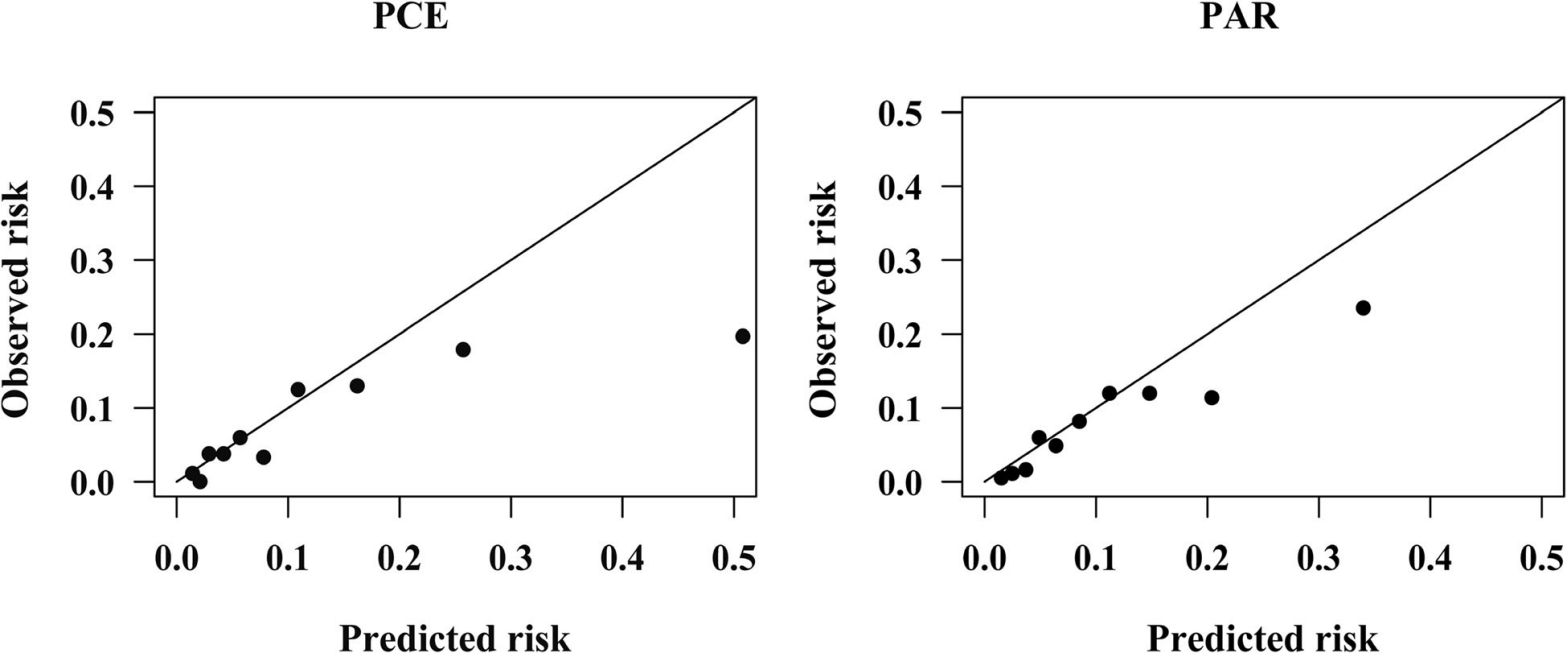
Calibration plots for the PCE and PAR in women after recalibration. PCE, Pooled Cohort Risk Equations; PAR, China-PAR risk equation; GND test chi-square statistics for risk equations: PCE: 92.83; *P* < 0.001. PAR: 26.17; *P* < 0.001

Recalibration did not change discriminations of risk equations, and calibrations of PCE and PAR in predicting the 5-year ASCVD risk slightly improved after recalibration in men, as shown in Fig. 4 and Fig. 5**.** After recalibration, the GND test χ^2^ for PAR was 3.47 (*P* = 0.628). The calibration of PCE in women improved but was still poor. Calibration plots in Fig. 5 showed that the overestimation of PCE and PAR in women mainly occurred in the top two deciles after recalibration, in which subjects had a 5-year estimated risk of ≥5%.

### Risk reclassification

Reclassification tables were constructed using clinically useful cutoff points of 2.5 and 5%. These tables show the numbers of women and men in our cohort who would be reclassified into the different risk groups while using different risk equations. For example, as shown in Table 3, with PCE, 53 (14.52%) of 365 women with a PAR risk of 2.5–5% would be reclassified as having ≥5% risk and 128 (35.07%) would be reclassified as having < 2.5% risk among women without ASCVD events. Of 255 women with PAR risk of < 2.5%, 28 (10.98%) were incorrectly reclassified into higher risk categories, and 127 (13.35%) of 951 women with FRS risk of ≥5% were correctly reclassified into lower categories among women without ASCVD events, resulting in a negative nonevent NRI of − 0.108 (− 0.131, − 0.088). In women with ASCVD events, 3 subject was correctly upgraded to higher-risk groups and 8 of them were incorrectly downgraded to lower-risk groups, leading to an event NRI of 0.033 (− 0.008, 0.080); thus, the total NRI was − 0.075(− 0.123, − 0.025), which indicated that using PCE, 7.5% of subjects would be correctly reclassified compared with PAR. Compared with the PAR, the PCE showed improved reclassification based on the NRI and cNRI in women. IDI showed no difference between PCE and PAR (− 0.005, 95% CI: − 0.018-0.013).

**Table 3:** Reclassification table comparing the recalibrated-PCE to the recalibrated-PAR to predict 5-year risk of ASCVD

PCE	PAR			Column total	N(%) of reclassified	NRI	cNRI	IDI
	Low	Moderate	High					
**Women**								
**Nonevent (*n*** **= 1571)**						−0.108 (−0.131, − 0.088)	−0.071 (− 0.119, − 0.023)	0.001(− 0.011, 0.008)
Low	227	128	8	363	136 (37.47)			
Moderate	23	184	119	326	142 (43.56)			
High	5	53	824	882	58 (6.58)			
Row total	255	365	951	1571	336 (21.39)			
**Event (*n*** **= 149)**						0.033 (−0.008, 0.080)	−0.339 (−0.495, −0.191)	−0.004 (− 0.010, 0.001)
Low	2	1	0	3	1 (33.33)			
Moderate	0	7	7	14	7 (50.00)			
High	0	3	129	132	3 (2.27)			
Row total	2	11	136	149	11 (7.38)			
Total						−0.075(−0.123, −0.025)	−0.409 (−0.584, −0.256)	− 0.005 (− 0.018, 0.013)
**Men**								
**Nonevent (*n*** **= 1339)**						0.093 (0.070,0.117)	0.448 (0.399, 0.497)	−0.001 (−0.013, 0.019)
Low	205	34	2	241	36 (14.94)			
Moderate	62	128	37	227	99 (43.61)			
High	13	120	738	871	133 (15.27)			
Row total	280	282	777	1339	268 (20.01)			
**Event (*n*** **= 137)**						−0.069(−0.125,-0.019)	−0.441(−0.578, −0.300)	0.023 (−0.005, 0.031)
Low	4	1	1	6	2 (33.33)			
Moderate	2	2	1	5	3 (60.00)			
High	0	13	113	126	13 (10.32)			
Row total	6	16	115	137	18 (13.14)			
Total						0.025 (−0.036,0.077)	0.006(−0.147, 0.166)	0.024 (0.001, 0.051)

Abbreviations: *PCE* Pooled Cohort Risk Equations, *PAR* China-PAR risk equation

NRI: net reclassification improvement; cNRI: continuous net reclassification improvement; IDI: integrated discrimination improvement

### Decision curve analysis (DCA)

**Fig. 6:**
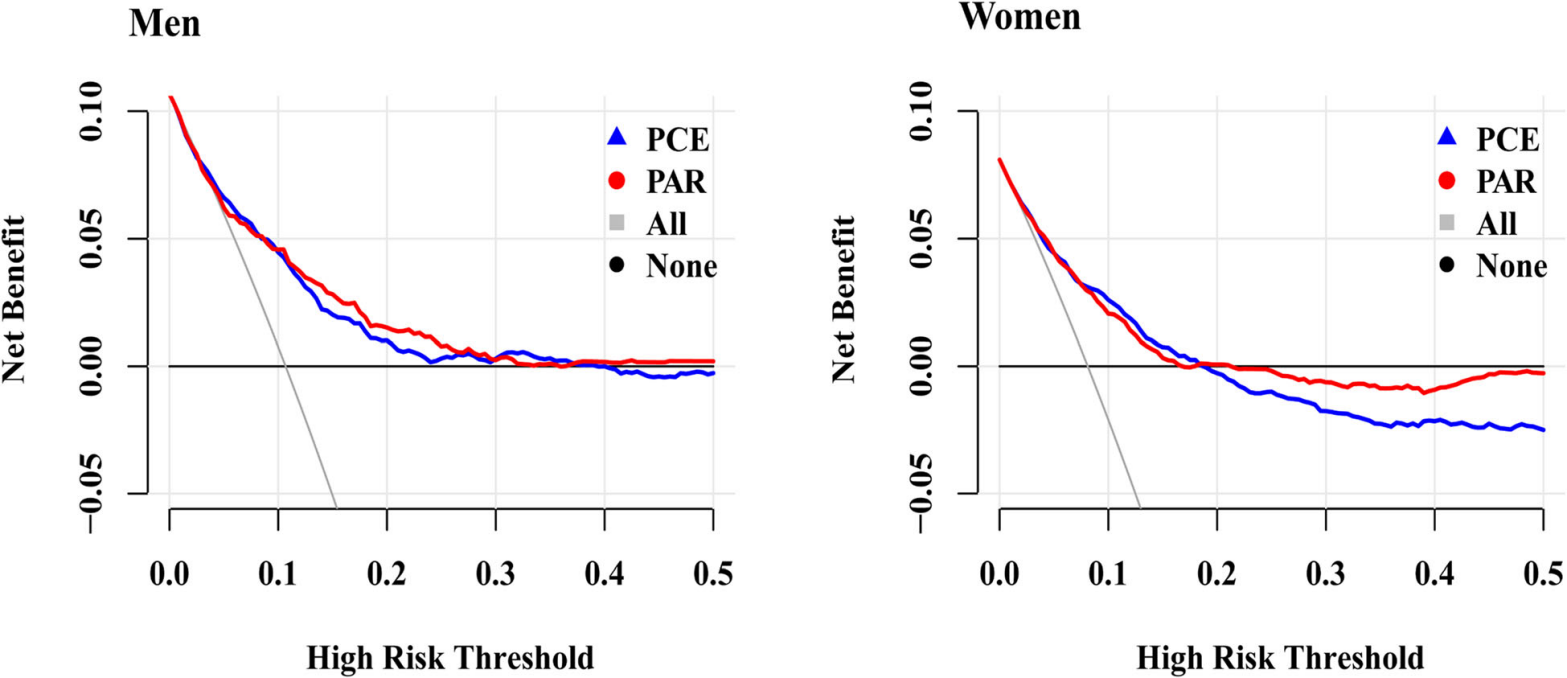
Decision curves for the PCE and PAR after recalibration. PCE, Pooled Cohort Risk Equations; PAR, China-PAR risk equation

We conducted DCA to compare the clinical usefulness of the three risk equations in predicting the 5-year ASCVD risks after recalibration, as shown in Fig. 6 and Additional file 1: Table S3. At the threshold risk of 5% among men, the net benefits for the PCE and PAR were 0.014, 0.010 greater than the values obtained by assuming positive in all subjects. This indicated that 27, 19 additional true-positive ASCVD events per 100 subjects can be correctly predicted without an increase in the number of false-positive results using the PCE and PAR, respectively. Similarly, in women, the net benefits were 0.043 and 0.042 for the PCE and PAR, respectively. The net benefits of PCE, PAR and FRS before recalibration were presented in Additional file 1: Table S4**.**

## Discussion

We conducted this study to externally validate the PAR equations newly recommended by the guideline and compared the performance of PAR against FRS and PCE in predicting the 5-year ASCVD risk both directly and after recalibration. The discrimination abilities in predicting the 5-year ASCVD risk for all three risk equations were moderate in both men and women of this cohort. All three risk equations systematically underestimated the 5-year ASCVD risk across nearly all ten deciles and were poorly calibrated for the outcome of ASCVD in women. The PAR had a relatively better calibration than the PCE and FRS, with 23% underestimation in women. The PAR and FRS fairly predicted the risk in men with a P/O of 1.05 and 1.06, respectively, whereas the PCE underestimated it by nearly 50% and was poorly calibrated. The results from comparisons of three risk equations based on the NRI, cNRI, and NRI statistics were not exactly consistent with those results measured by C-indices. For example, the FRS (0.761) had a higher C index than PCE (0.738) in women, however, the results based on NRI, cNRI and IDI suggested that PCE had a better reclassification ability than FRS. A study [23] suggested that the NRI and IDI could be strongly affected by miscalibration of a model, and the PCE and PAR in women and the PCE in men were still poorly calibrated after recalibration; thus, the results may be affected.

The PCE, released by the American College of Cardiology and the American Heart Association in 2013, has undergone numerous external validations in various populations and settings. Most results from external validation studies indicated that the PCE tended to overestimate ASCVD risk [11, 24–27]. For example, Muntner et al. [11] found that the PCE overestimated the 5-year ASCVD risk in the overall population and demonstrated that the overestimation may be explained by the lack of active ASCVD surveillance and the high prevalence of statin use in contemporary cohorts. A study conducted in a large contemporary, multiethnic population suggested that the PCE substantially overestimated the observed 5-year risk and was poorly calibrated [24]. Similar results from the PREDICT study in New Zealand showed that the PCE systematically overestimated the ASCVD risk by approximately 40% in men and 60% in women [25].

Because the Asian population was excluded in the PCE’s derivation cohorts, there is substantial overestimation when PCE was externally validated in the Asian population [28–30]. The PCE had moderate discrimination and overestimated the 10-year ASCVD risk in the Malaysian population. The researchers suggested that the overestimation could be explained by the treatment during the 10-year follow-up and observed that the proportion of patients receiving statin therapy increased from 9.7 to 63.7% in the 10-year period [28]. The Hong Kong Cardiovascular Risk Factor Prevalence Study [30] also found that the PCE was poorly calibrated for men and had moderate discrimination in both men and women.

Most importantly, the possible reasons for the overestimation of PCE may be the under-ascertainment of ASCVD events, increase in statin use, decline in ASCVD incidence rates over time, and different ethnic backgrounds [11, 26, 28].

However, the PCE systematically underestimated risks both in men and women in this population, similar to the results from several studies [5, 31–33]. The Korean Heart Study reported that the PCE (White model) overestimated the 10-year ASCVD risk by 56.5% in men but underestimated it by 27.9% in women. They suggested that the varying rates of ASCVD incidence and associated risk factors from the derivation cohorts of PCE may be the reason that the PCE did not calibrate well in their study [29]. The results from the Austrian health-screening program demonstrated that the PCE underestimated 5-year ASCVD risk over the whole range of predicted risks and P/O ratios were 0.54 for women and 0.73 for men [31]. The PCE systematically underpredicted the 5-year ASCVD risks in subjects from disadvantaged communities in Cleveland, and researchers concluded that the poor calibration of the PCE in disadvantaged communities could be explained by the variations in ASCVD risk factors and environmental and other neighborhood-level exposures across the socioeconomic spectrum [32]. Similar results from the REGARDS study revealed that the PCE underestimated ASCVD risk in subjects with more social deprivation but overestimated ASCVD risk in subjects with less social deprivation [33]. Subjects in this external validation population were from disadvantaged rural areas in northwestern China and more deprived than individuals from other areas in China.

The PAR, derived from the general Chinese population, had moderate discrimination and was well calibrated in men but substantially underestimated the 5-year ASCVD risk in women in this study. However, a recently published study conducted in a rural northern Chinese population indicated that the PAR fairly predicted the 5-year ASCVD risk in men but overestimated it by 29.4% in women [5].

Although FRS was designed to predict a broader endpoint of global CVD, it underpredicted the 5-year risk in women. Nevertheless, the FRS slightly overestimated the risk in men with a P/O of 1.06 and was better calibrated.

Underestimation of the 5-year ASCVD risk in women by three risk equations could be explained by several aspects. Xinjiang is on the “stroke belt,” and the estimated age-standardized incidence of stroke in Xinjiang per 100,000 persons is 191 [34], higher than that in the United States (140 per 100,000) [35]. The incidence of ASCVD is close between men and women in this population. Thus, the higher incidence rates of ASCVD in China than that in the USA may be the reason why the PCE and FRS, derived from the US population, underestimated the ASCVD risk in women. Researchers have shown that social deprivation is associated with high ASCVD incidence rates [36, 37]. Most Kazakh and Uygur individuals live in a typical low-income and multi-ethnic rural area in northwest China, Xinjiang, where most live on $1.00 or less. The prevalence rate of dyslipidemia and hypertension in this population is relatively higher than that in the Han nationality with low awareness, treatment, and control rates due to their genetic backgrounds and unique dietary habits [38, 39], so the ASCVD incidence rates in this population are relatively high [40]. This may partly explain why the PAR, developed in the general Chinese population, underestimated the 5-year ASCVD risk of women in this population. Another potential reason for ASCVD risk underestimation by three risk equations is that the cohort in this study is more contemporary than the derivation cohorts of these two risk equations, and the quantitative relationship between risk factors and ASCVD events may have changed with time. The study population had a much lower level of TC and a high level of HDL than that in the derivation population of three risk equations, so risks predicted by using the coefficients from three risk equations might be lower. These factors might explain, to some extent, the findings of risk underestimation by the PCE and PAR in women.

Recalibration is necessary before applying a risk equation to a specific population which is excluded in the risk equation’s derivation cohorts and allows us to directly compare three risk equations using reclassification tables. Therefore, we recalibrated the PCE and PAR to our cohorts using the method proposed by D’Agostino et al. [18]. However, there was an overestimation of ASCVD risk after recalibration, especially in women, which may be explained by that recalibration would not change the coefficients between risk factors and ASCVD outcome, but risk factors in this population were also different from those cohorts and the incidence rate of ASCVD in this population was higher than that in the derivation cohort of three risk equations. More details can be seen in Additional file 1: Table S5**.** Improved calibration is of vital importance to risk classification and evaluation of net benefit, so recalibration can be a useful method to tailor and generalize a risk equation to an external population [41].

DCA was performed to investigate the clinical usefulness of the three equations. At the recommended threshold risk of 5% for the 5-year ASCVD risk, the most useful risk equation was the PCE for both men and women at the threshold probability of 5% after recalibration. A higher risk threshold will leave more high-risk subjects untreated, and a lower threshold will lead to more unnecessary treatments. Thus, it is important to set an accurate and appropriate threshold to identify high-risk subjects to balance the benefits and risks of recommended therapies and guide decision making in practice.

### Strengths and limitations

One strength of our study is the complete ascertainment of ASCVD events using health insurance claims, patients’ hospital medical records, and questionnaire responses; this may partly explain the risk underestimation by the PCE and PAR in women. Moreover, this population is representative, and the results can be generalized to the entire Kazakh and Uygur populations.

Nevertheless, some limitations merit explanations. The main limitation is that the sample size of our study is relatively small. Another limitation is that this study is ongoing and the follow-up period is < 10 years, so we can only calculate the 5-year ASCVD risk. The baseline survival of FRS was calculated based on 10-year Kaplan-Meier CVD rate, which was different from PCE and PAR. This may cause miscalibration of three equations, which were designed to predict 10-year ASCVD risk. A future study is needed to ensure their accurate calibrations over a longer period.

## Conclusions

Therefore, we have externally validated three risk equations in predicting the 5-year ASCVD risk in an underrepresented population from rural areas in northwestern China. Results indicated that all three risk equations have moderate discrimination in both men and women. However, they underestimated the ASCVD risk for women. The PAR, derived from Chinese cohorts, had moderate discrimination and was well calibrated in men but underestimated the risk in women. After recalibration, the PAR had the most accurate predictions. The PCE had better reclassification than PAR. DCA indicated that, at the threshold risk of 5%, the most clinically useful risk equation was PCE in both men and women after recalibration. Indicators used to examine predictive performances of three risk equations showed inconsistent results, none of them is suitable for direct application in this population, even after recalibration. Future ASCVD risk equations special for this Kazakh and Uygur population are required in the health screening program for the primary prevention of ASCVD.

## Supplementary information

**Additional file 1 Table S1.** Parameters of three risk equations used in this study for men and women. **Table S2.** Summary of risk factors and outcome of three risk equations used in this external validation study. **Table S3.** Net benefit analysis for preventing ASCVD at different thresholds of Pt after recalibration. **Table S4.** Net benefit analysis for preventing ASCVD at different thresholds of Pt before recalibration. **Table S5.** Comparisons of baseline characteristics between three risk equations and the study population.

**Additional file 2 Fig. S1.** Distribution of risk estimated from the PCE, PAR, and FRS among men and women.

## Data Availability

The datasets used during the current study are available from the corresponding author on reasonable request. The Chinese questionnaire copy may be requested from the authors.

## Abbreviations

ACC: American College of Cardiology
AHA: American Heart Association
ASCVD: Atherosclerotic Cardiovascular Disease
CHD: Coronary Heart Disease
CVD: Cardiovascular Disease
cNRI: continuous Net Reclassification Improvement
DBP: Diastolic Blood Pressure
DCA: Decision Curve Analysis
FRS: Framingham Risk Score
GND: Greenwood-Nam-D’Agostino
HDL-C: High Density Lipoprotein Cholesterol
IDI: Integrated Discrimination Improvement
PAR: China-Population Attributable Risk equations
PCE: Pooled Cohort Equations
SBP: Systolic Blood Pressure
SD: Standard Deviation
NRI: Net Reclassification Improvement
