# Supplementary material for: External validation of three atherosclerotic cardiovascular disease risk equations in rural areas of Xinjiang, China
Source: BMC Public Health. 2020 Sep 29;20:1471. doi: 10.1186/s12889-020-09579-4 (PMC7526265; doi:10.1186/s12889-020-09579-4)
Supplement: Supplementary file 1 — Additional file 1 Table S1. Parameters of three risk equations used in this study for men and women. Table S2. Summary of risk factors and outcome of three risk equations used in this external validation study. Table S3. Net benefit analysis for preventing ASCVD at different thresholds of Pt after recalibration. Table S4. Net benefit analysis for preventing ASCVD at different thresholds of Pt before recalibration. Table S5. Comparisons of baseline characteristics between three risk equations and the study population. [file 12889_2020_9579_MOESM1_ESM.docx]

**Tables**

| **Table S1. Parameters of three risk equations used in this study for men and women** | | | |
| --- | --- | --- | --- |
| **Model** | **S_0_(t) at 5 years** | **Mean Score** | **Equations for PCE, PAR and FRS** |
| **Men** | | | |
| PCE (White Model) | 0.9625 ^a^ | 61.18 | =12.344×ln(age)+11.853×ln(TC)-2.664×ln(age)×ln(TC)-7.990×ln(HDLC)+1.769×ln(age)×ln(HDLC) (+1.797×ln(SBP) if hypertension treated) (+1.764×ln(SBP) if hypertension untreated) (+7.837-1.795×ln(age) if current smoker) (+0.658 if diabetes) |
| PAR | 0.9724^b^ | 140.68 | =31.97×ln(age)+0.62×ln(TC)-0.69×ln(HDLC)-0.71×ln(waist) (+[27.39-6.02×ln(age)]×ln(SBP) if hypertension treated) (+[26.15-5.73×ln(age)]×ln(SBP) if hypertension untreated) (+3.96-0.94×ln(age) if current smoker) (+6.22-1.53×ln(age) if having family history of ASCVD) (+0.36 if diabetes) (+0.48 if in Northern China) (-0.16 if living in urban) |
| FRS | 0.9431^c^ | 23.98 | =3.0617×ln(age)+1.12370×ln(TC)-0.93263×ln(HDLC) (+1.93303×ln(SBP) if hypertension untreated) (+1.99881×ln(SBP) if hypertension treated) (+0.65451 if current smoker) (+0.57367 if diabetes) |
|  | | | |
| **Women** | | | |
| PCE (White Model) | 0.989 ^a^ | -29.18 | =-29.799×ln(age)+4.884×ln(age)×ln(age)+13.540×ln(TC)-3.114×ln(age)×ln(TC)-13.578×ln(HDLC)+3.149×ln(age)×ln(HDLC) (+2.019×ln(SBP) if hypertension treated) (+1.957×ln(SBP) if hypertension untreated) (+7.574-1.665×ln(age) if current smoker) (+0.661 if diabetes) |
| PAR | 0.9841^b^ | 117.26 | =24.87×ln(age)+0.06×ln(TC)-0.22×ln(HDLC)+1.48×ln(waist) (+[20.71-4.53×ln(age)] ×ln(SBP) if hypertension treated) (+[19.98 -4.36×ln(age)]×ln(SBP) if hypertension untreated) (+0.49 if current smoker) (+0.57 if diabetes) (+0.54 if in Northern China) |
| FRS | 0.9747^c^ | 26.193 | =2.32888×ln(age)+1.20904×ln(TC)-0.70833×ln(HDLC) (+1.76157×ln(SBP) if hypertension untreated) (+2.82263×ln(SBP) if hypertension treated) (+0.52873 if current smoker) (+0.69154 if diabetes) |
| ^a^ Obtained from the supplementary files of Muntner, et al (JAMA 2014).  ^b^ S_0_(t)=exp (-5-year Kaplan-Meier ASCVD incidence rate)  ^c^ S_0_(t)=exp (-10-year Kaplan-Meier CVD incidence rate/2) | | | |

| **Table S2. Summary of risk factors and outcome of three risk equations used in this external validation study.** | | | | |
| --- | --- | --- | --- | --- |
| Risk score | Derivation cohorts | Outcome | Risk factors | Internal validation C statistics |
| ASCVD Pooled Cohort Risk Equations(PCE) | Atherosclerosis Risk in Communities(ARIC) Cardiovascular Health Study(CHS) Coronary Artery Risk Development in Young Adults(CARDIA) Framingham Original and Offspring cohort | A first ASCVD event, defined as nonfatal myocardial infarction(MI) or coronary heart disease(CHD) death, or fatal or nonfatal stroke | Age(40-79 years) Sex(women/men) Treated or untreated systolic blood pressure(mmHg) Total cholesterol (mg/dL) High-density lipoprotein cholesterol(mg/dL) Current smoking(Yes/No) Diabetes(Yes/No) | White women:0.8040(0.025) White men:0.7443(0.023) African American women:0.8142(0.037) African American men:0.7036(0.051) |
|  |  |  |  |  |
|  |  |  |  |  |
|  |  |  |  |  |
|  |  |  |  |  |
|  |  |  |  |  |
|  |  |  |  |  |
| The China-PAR risk equation(PAR) | The International Collaborative Study  of Cardiovascular Disease in Asia(InterASIA)  The China Multi-Center Collaborative Study of Cardiovascular(China MUCA 1998) | ASCVD was defined as nonfatal acute MI or CHD death, or fatal or nonfatal stroke | Age(35-74 years) Sex(women/men) Waist circumference (cm) Treated or untreated systolic blood pressure(mmHg) Total cholesterol (mg/dL) High-density lipoprotein cholesterol(mg/dL) Current smoking(Yes/No) Diabetes(Yes/No) Geographic region(Northern China/southern China) Urbanization(Urban/Rural)^*^ Family History of ASCVD(Yes/No)^*^ | Men:0.794(0.775,0.814) Women:0.811(0.787,0.835) |
|  |  |  |  |  |
|  |  |  |  |  |
|  |  |  |  |  |
|  |  |  |  |  |
|  |  |  |  |  |
|  |  |  |  |  |
|  |  |  |  |  |
|  |  |  |  |  |
|  |  |  |  |  |
|  |  |  |  |  |
| Framingham Risk Score 2008 (FRS) | The original Framingham Heart Study The Framingham Offspring Study | CVD was defined as a composite of CHD (coronary death, myocardial infarction, coronary insufficiency, and angina), cerebrovascular events (including ischemic stroke, hemorrhagic stroke, and transient ischemic attack), peripheral artery disease (intermittent claudication), and heart failure. | Age(40-79 years) Sex(women/men) Treated or untreated systolic blood pressure(mmHg) Total cholesterol (mg/dL) High-density lipoprotein cholesterol(mg/dL) Current smoking(Yes/No) Diabetes(Yes/No) | Men:0.763(0.746,0.780) Women:0.793(0.772,0.814) |
| Abbreviations: CVD, Cardiovascular Disease; ASCVD, Atherosclerotic Cardiovascular Disease; CHD, Coronary Heart Disease  ^*^ These risk factors were not included in the PAR model for women. | | | | |

| **Table S3. Net benefit analysis for preventing ASCVD at different thresholds of Pt** **after recalibration.** | | | | | | | | | | | | |
| --- | --- | --- | --- | --- | --- | --- | --- | --- | --- | --- | --- | --- |
| Risk equations | Pt (%) | **Women** | | | | |  | **Men** | | | | |
|  |  | Net Benefit | | |  | Net Benefit ^a^ |  | Net Benefit | | |  | Net Benefit ^a^ |
|  |  | Treat all |  | Prediction model |  |  |  | Treat all |  | Prediction model |  |  |
| PCE | 2.5 | 0.035 |  | 0.046 |  | 0.012 |  | 0.068 |  | 0.074 |  | 0.007 |
|  | 5 | -0.019 |  | 0.024 |  | 0.043 |  | 0.043 |  | 0.057 |  | 0.014 |
|  | 10 | -0.146 |  | -0.001 |  | 0.145 |  | -0.010 |  | 0.035 |  | 0.046 |
|  |  |  |  |  |  |  |  |  |  |  |  |  |
| PAR | 2.5 | 0.035 |  | 0.046 |  | 0.012 |  | 0.068 |  | 0.075 |  | 0.008 |
|  | 5 | -0.019 |  | 0.023 |  | 0.042 |  | 0.043 |  | 0.053 |  | 0.010 |
|  | 10 | -0.146 |  | 0.002 |  | 0.149 |  | -0.010 |  | 0.036 |  | 0.046 |
| Abbreviations: PCE, Pooled Cohort Risk Equations; PAR, China-PAR risk equation | | | | | | | | | | | | |
| Pt: threshold probability in %;  ^a^ Net benefit of using risk equations compared with treating all subjects | | | | | | | | | | | | |

| Table S**4**. Net benefit analysis for preventing ASCVD at different thresholds of Pt before **recalibration**. | | | | | | | | | | | | |
| --- | --- | --- | --- | --- | --- | --- | --- | --- | --- | --- | --- | --- |
| Risk equations | Pt (%) | **Women** | | | | |  | **Men** | | | | |
|  |  | Net Benefit | | |  | Net Benefit ^a^ |  | Net Benefit | | |  | Net Benefit ^a^ |
|  |  | Treat all |  | Prediction model |  |  |  | Treat all |  | Prediction model |  |  |
| PCE | 2.5 | 0.057 |  | 0.054 |  | -0.004 |  | 0.068 |  | 0.074 |  | 0.006 |
|  | 5 | 0.033 |  | 0.033 |  | 0.000 |  | 0.043 |  | 0.048 |  | 0.005 |
|  | 10 | -0.021 |  | 0.012 |  | 0.033 |  | -0.010 |  | 0.017 |  | 0.027 |
|  |  |  |  |  |  |  |  |  |  |  |  |  |
| PAR | 2.5 | 0.057 |  | 0.058 |  | 0.001 |  | 0.068 |  | 0.075 |  | 0.007 |
|  | 5 | 0.033 |  | 0.032 |  | -0.001 |  | 0.043 |  | 0.053 |  | 0.010 |
|  | 10 | -0.021 |  | 0.013 |  | 0.034 |  | -0.010 |  | 0.035 |  | 0.045 |
|  |  |  |  |  |  |  |  |  |  |  |  |  |
| FRS | 2.5 | 0.057 |  | 0.057 |  | 0.000 |  | 0.068 |  | 0.074 |  | 0.006 |
|  | 5 | 0.033 |  | 0.037 |  | 0.004 |  | 0.043 |  | 0.058 |  | 0.015 |
|  | 10 | -0.021 |  | 0.015 |  | 0.036 |  | -0.010 |  | 0.037 |  | 0.047 |
| Abbreviations: PCE, Pooled Cohort Risk Equations; PAR, China-PAR risk equation; FRS, Framingham Risk Score 2008 | | | | | | | | | | | | |
| Pt: threshold probability in %; | | | | | | | | | | | | |
| ^a^ Net benefit of using risk equations compared with treating all subjects | | | | | | | | | | | | |

| **Table S5. Comparisons of baseline characteristics between three risk equations and the study population** | | | | | | | | | | | |
| --- | --- | --- | --- | --- | --- | --- | --- | --- | --- | --- | --- |
| **Baseline Characteristics** | **Population** | | | | | | | | | | |
|  | **PCE** | |  | **FRS** | |  | **PAR** | |  | **Validation population** | |
| **Women** | | | | | | | | | | | |
| Age (yrs) | 56.8 | 9.5 |  | 49.1 | 11.1 |  | 48.4 | 9.2 |  | 51.1 | 8.9 |
| Total Cholesterol (mg/dl) | 220.5 | 41.9 |  | 215.1 | 44.1 |  | 188.2 | 38.3 |  | 184.9 | 33.5 |
| HDL Cholesterol (mg/dL) | 58.2 | 16.4 |  | 57.6 | 15.3 |  | 53.4 | 12.8 |  | 50.9 | 11.8 |
| Untreated SBP (mmHg) | 121.1 | 19.2 |  | 125.8 | 20.0 |  | 122.5 | 20.6 |  | 131.3 | 23.1 |
| Treated SBP (mmHg) | 136.9 | 20.8 |  |  |  |  |  |  |  |  |  |
| Waist Ciecumference(cm) |  |  |  |  |  |  | 76.5 | 9.7 |  | 84.3 | 10.7 |
| BP Meds (%) | 18.5 |  |  | 11.76 |  |  | 7.4 |  |  | 13.9 |  |
| Current Smoker (%) | 24.9 |  |  | 34.23 |  |  | 4.5 |  |  | 12.9 |  |
| Diabetes (%) | 6.3 |  |  | 3.76 |  |  | 5.1 |  |  | 6.6 |  |
| Family History of ASCVD |  |  |  |  |  |  | 13.0 |  |  | 3.6 |  |
|  | | | | | | | | | | | |
| **Men** | | | | | | | | | | | |
| Age (yrs) | 51.6 | 9.1 |  | 48.5 | 10.8 |  | 48.8 | 9.4 |  | 53.8 | 9.4 |
| Total Cholesterol (mg/dl) | 193.4 | 38.3 |  | 212.5 | 39.3 |  | 186.6 | 37.6 |  | 183.4 | 33.3 |
| HDL Cholesterol (mg/dL) | 40.7 | 12.5 |  | 44.9 | 12.2 |  | 50.4 | 13.9 |  | 49.1 | 9.8 |
| Untreated SBP (mmHg) | 113.8 | 17.5 |  | 129.7 | 17.6 |  | 124.8 | 19.0 |  | 134.3 | 22.6 |
| Treated SBP (mmHg) | 124.9 | 20.4 |  |  |  |  |  |  |  |  |  |
| Waist Ciecumference(cm) |  |  |  |  |  |  | 80.1 | 10.0 |  | 87.0 | 10.2 |
| BP Medications (%) | 15.5 |  |  | 10.13 |  |  | 5.8 |  |  | 3.3 |  |
| Current Smoker (%) | 23.4 |  |  | 35.22 |  |  | 60.7 |  |  | 41.9 |  |
| Diabetes (%) | 8.1 |  |  | 6.5 |  |  | 5.1 |  |  | 9.5 |  |
| Family History of ASCVD |  |  |  |  |  |  | 13.4 |  |  | 3.18 |  |
| Abbreviations: SD, Standard Deviation; SBP, systolic blood pressure; DBP, diastolic blood pressure; HDL-C, high density lipoprotein cholesterol; ASCVD, Atherosclerotic Cardiovascular Disease; PCE, Pooled Cohort Risk Equations; PAR, China-PAR risk equation; FRS, Framingham Risk Score 2008 | | | | | | | | | | | |
