# Supplementary material for: External validation of three atherosclerotic cardiovascular disease risk equations in rural areas of Xinjiang, China
Source: BMC Public Health. 2020 Sep 29;20:1471. doi: 10.1186/s12889-020-09579-4 (PMC7526265; doi:10.1186/s12889-020-09579-4)
Supplement: Supplementary file 2 — Additional file 2 Fig. S1. Distribution of risk estimated from the PCE, PAR, and FRS among men and women. [file 12889_2020_9579_MOESM2_ESM.docx]

**Figure legends**

**Fig. S1. Distribution of risk estimated from the PCE, PAR, and FRS among men and women.**

PCE, Pooled Cohort Risk Equations; PAR, China-PAR risk equation; FRS, Framingham Risk Score 2008

| 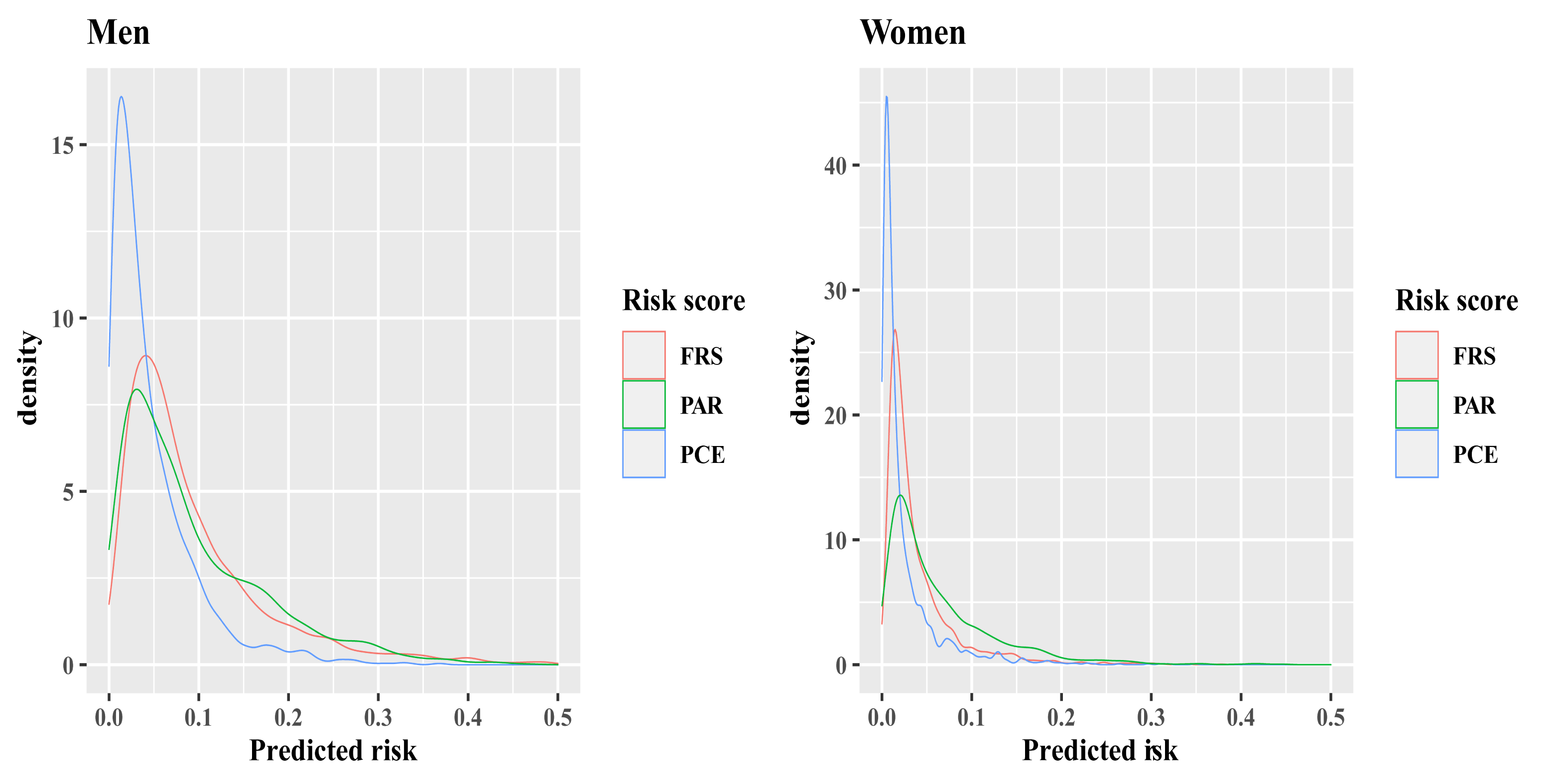 |
| --- |
| **Fig. S1. Distribution of risk estimated from the PCE, PAR, and FRS among men and women.**  PCE, Pooled Cohort Risk Equations; PAR, China-PAR risk equation; FRS, Framingham Risk Score 2008; |
